# Parasitic leiomyoma of the small bowel mesentery mimicking a gastrointestinal stromal tumor: a case report

**DOI:** 10.3389/fonc.2026.1788561

**Published:** 2026-06-02

**Authors:** Shangqin Lin, Mingzhe Hu, Zhifeng Xu, Wei Weng, Yaomeng Chen

**Affiliations:** 1 Department of Radiology, Wenzhou People’s Hospital, Wenzhou, Zhejiang, China; 2 Department of General Surgery, Wenzhou People’s Hospital, Wenzhou, Zhejiang, China; 3 Department of Radiology, The Third Affiliated Hospital of Wenzhou Medical University, Wenzhou, Zhejiang, China

**Keywords:** case report, gastrointestinal stromal tumor (GIST), iatrogenic, laparoscopic myomectomy, parasitic leiomyoma

## Abstract

**Background:**

Parasitic leiomyoma is a rare subtype of uterine leiomyoma located outside the uterus, often deriving its blood supply from adjacent organs. With the popularization of laparoscopic surgery, reports of iatrogenic parasitic leiomyoma (IPL) as a long-term complication of unconfined morcellation have been increasingly frequent. These tumors may mimic gastrointestinal tumors, posing a significant challenge to clinical diagnosis.

**Case description:**

A 38-year-old female presented with periumbilical pain accompanied by abdominal distension for 1 week. The patient had a history of laparoscopic uterine myomectomy 6 years ago. Abdominal CT suggested a mass in the small bowel mesentery. This lesion was initially suspected to be a small bowel gastrointestinal stromal tumor (GIST). The patient underwent laparoscopy, during which a solid tumor attached to the small bowel mesentery with an independent vascular pedicle was found. The mass was surgically resected, and histopathological examination confirmed it as a parasitic uterine leiomyoma with red degeneration. The patient recovered smoothly after surgery.

**Conclusion:**

This case highlights the difficulty in differentiating parasitic uterine leiomyomas from gastrointestinal tumors based solely on imaging features. For patients with a history of uterine surgery (particularly those who underwent laparoscopic myomectomy with a history of morcellation), parasitic uterine leiomyoma should be included in the differential diagnosis if an abdominal mass is detected. Early recognition helps to avoid unnecessary extensive bowel resection, thereby preventing unnecessary radical resection.

## 1 Introduction

Uterine leiomyomas are the most common benign monoclonal tumors in women of reproductive age. Although typically confined to the uterus, rare variants located within the peritoneal cavity and separated from the uterus occasionally occur, known as parasitic uterine leiomyomas. This disease was first described by Kelly and Cullen in 1909, who at the time believed it originated from a pedunculated subserosal myoma that acquired a new blood supply from adjacent tissues after losing its connection to the uterus (1).

However, the epidemiological characteristics of the disease have shifted. Recent evidence suggests that most modern cases are iatrogenic, originating from the dissemination of tissue fragments during laparoscopic myomectomy, particularly when power morcellation is performed without using a specimen bag (2, 3). A recent retrospective analysis by Murakami et al. further confirmed the significant association between morcellation and the subsequent development of parasitic uterine leiomyomas (4). Parasitic uterine leiomyomas can implant onto various peritoneal surfaces, including the intestines, bladder, and greater omentum (5), leading to diverse clinical manifestations that are often misdiagnosed as ovarian malignancies or gastrointestinal tumors (6, 7). Herein, we report a case of parasitic uterine leiomyoma attached to the small bowel mesentery of a 38-year-old female, which was initially diagnosed clinically as a small bowel gastrointestinal stromal tumor (GIST).

## 2 Case report

A 38-year-old female patient was admitted to the hospital due to “periumbilical pain accompanied by abdominal distension for 1 week”. The pain was mild, without radiation, and was not accompanied by fever, nausea, or vomiting. In 2019, the patient underwent a laparoscopic uterine myomectomy for “uterine fibroids”. She denied any history of hypertension, diabetes, or other chronic systemic diseases.

Physical examination revealed a flat abdomen, mild periumbilical tenderness, no palpable mass, and no rebound tenderness. Tumor markers (including CA-125, CA19-9, CEA, and AFP) were all within normal ranges.

Abdominal CT scan showed a mass-like soft tissue density shadow in the small bowel mesentery region, measuring approximately 43 mm × 58 mm. The lesion had clear boundaries and a well-defined demarcation from the adjacent small bowel loops, with no obvious signs of bowel wall infiltration. After contrast enhancement, the lesion showed heterogeneous enhancement, with the solid portion showing significant enhancement and low-enhancement necrotic areas visible inside (**Figures 1A–D**). Based on the imaging features, anatomical location, and the incidence of primary mesenchymal tumors of the mesentery, the preliminary diagnosis was gastrointestinal stromal tumor (GIST) with potential malignant transformation.

**Figure 1:**
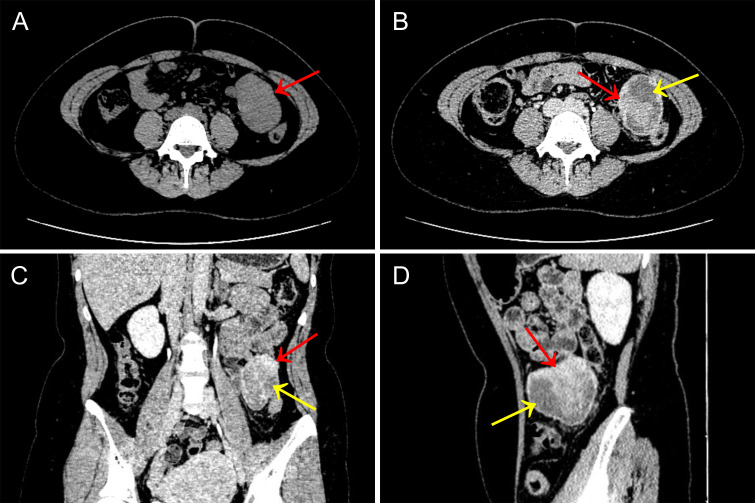
**(A)** Axial CT image shows a mass-like soft tissue density shadow (red arrow) in the small bowel mesentery area of the left lower abdomen, with clear boundaries, measuring approximately 43 mm × 58 mm. **(B–D)** Enhanced axial, coronal, and sagittal CT images show heterogeneous enhancement of the mass after contrast injection (the red arrow indicates the significantly enhanced area, and the yellow arrow indicates the low-enhancement necrotic area).

Given that imaging suggested a possible malignant tumor and the patient presented with persistent abdominal symptoms, the surgeons decided to perform surgical exploration and lesion resection. Intraoperatively, the mass was found deep in the small bowel mesentery, and the feeding vessels at the root of the tumor connected to the descending colon like a pedicle, providing an independent source of blood supply. The surgical team performed a laparoscopic mass resection, and all specimens were retrieved using an endoscopic retrieval bag.

Pathological HE staining showed that the tumor was composed of fascicular or interlacing long spindle-shaped cells with abundant eosinophilic cytoplasm. In some cells, the cytoplasm was preserved while the nuclei disappeared; concurrently, mitotic figures were rare, and no coagulative necrosis of tumor cells was observed (**Figure 2**). Immunohistochemical (IHC) staining further confirmed that the smooth muscle marker actin (ACT) was positive (+), whereas the GIST markers (CD117, DOG1) and CD10 were all negative (-). Furthermore, the Ki67 proliferation index was only 5%, indicating extremely low tumor proliferative activity. Combining the aforementioned histological morphology and immunophenotype, this case could definitively exclude malignant tumors such as GIST and leiomyosarcoma, and a final diagnosis of small bowel mesenteric leiomyoma with red degeneration was confirmed. The patient recovered smoothly postoperatively, and the pelvic drainage tube was removed on the 7th postoperative day. A review CT scan before discharge showed no residual mass, and the patient was discharged on the 8th day with instructions for outpatient follow-up.

**Figure 2:**
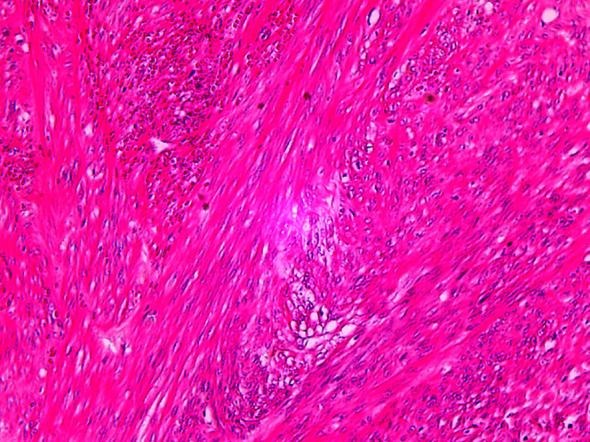
HE staining shows that the tumor is composed of fascicular or interlacing long spindle-shaped tumor cells with abundant eosinophilic cytoplasm; in some cells, the cytoplasm is present while the nucleus has disappeared, and mitotic figures are rare.

## 3 Discussion

Parasitic uterine leiomyoma is a rare solid tumor that accounts for only a small fraction of extrauterine myomas. A review by Salih et al. highlighted its rarity and diagnostic difficulty (8). Its pathogenesis is divided into primary (spontaneous) and secondary (iatrogenic). Primary cases are extremely rare, whereas the incidence of iatrogenic parasitic uterine leiomyoma has risen with the widespread use of laparoscopic uterine myomectomy and power morcellation (2, 5). Yasumi et al. reported a rare case of a spontaneously developed retroperitoneal parasitic myoma (9), while Ramdass et al. described a complex case accompanied by arteriovenous malformation, portal hypertension, and cardiac failure (10); these recent reports demonstrate the extreme heterogeneity of the clinical presentation of this disease. The patient in this case had undergone a myomectomy 6 years ago, which is consistent with the typical latent period of iatrogenic parasitic uterine leiomyoma (2 to 10 years postoperatively) (7).

The clinical manifestations of parasitic uterine leiomyoma are highly variable, depending on the site of implantation. Most patients are asymptomatic, but some can present with abdominal pain, abdominal distension, or compressive symptoms, as demonstrated in this case. Guerra et al. even reported a rare case located perihepatically, further broadening the anatomical scope we need to consider (11). When the tumor involves the intestines or mesentery, it easily mimics gastrointestinal malignancies such as GIST or carcinoma (6). In this case, the location of the mass in the small bowel mesentery, combined with its imaging characteristics of soft tissue density and heterogeneous enhancement, led to the initial misdiagnosis as a small bowel GIST. Ahmed et al. (12) and Barat et al. (13) noted that GISTs often appear on CT as well-defined masses but are frequently accompanied by heterogeneous enhancement due to hemorrhage, necrosis, or cystic degeneration, especially when the tumor size is large (14). A study by Maldonado et al. further emphasized that biologically aggressive GISTs more commonly present with central necrosis, ulceration, or surrounding infiltration (14). In contrast, Kang et al., when comparing schwannomas with GISTs, pointed out that benign mesenchymal tumors (such as leiomyomas) tend to exhibit homogeneous enhancement and fewer necrotic areas unless red degeneration occurs (15). As summarized in **Table 1**, these differential features are critical for clinical decision-making. However, due to the lack of specificity, Cohen et al. also noted that preoperative definitive diagnosis relying solely on CT imaging remains challenging (16).

**Table 1:** Comparison table for differential diagnosis of imaging and clinical features of abdominal tumors. .

Imaging and clinical features	Parasitic leiomyoma	Gastrointestinal stromal tumor (GIST)	Leiomyosarcoma (LMS)	Abdominal/mesenteric schwannoma
References	(6)	(17)	(18)	(15, 19)
Primary site of occurrence	Peritoneum, mesentery, greater omentum; usually separated from the uterine body	Stomach (60%), small bowel (30%); typically grows exophytitally from the intestinal wall	Retroperitoneum, mesentery, large vessels; exhibits infiltrative growth	Retroperitoneum, mesentery; distributed along the nerve course, with clear boundaries
CT enhancement pattern	Homogeneous enhancement when the lesion is small; heterogeneous enhancement when the lesion is large, accompanied by solid component enhancement	Heterogeneous enhancement; solid components show significant, patchy, or peripheral enhancement	Markedly heterogeneous enhancement, with ill-defined and infiltrative margins	Progressive, delayed, or mottled heterogeneous enhancement
Necrosis/degeneration	Rare; occurs only during red degeneration or myxoid degeneration, which can mimic malignant manifestations	Very common; can present with central necrosis, cystic change, or fistula formation	Extremely common; accompanied by significant central necrosis and hemorrhage	Common in larger tumors; typically presents as central cystic change
MRI signal features	High T1WI signal (during red degeneration); low T2WI signal (solid smooth muscle component)	Long T1, long T2 signals (cystic fluid component); highly heterogeneous T1/T2 signals	Intermediate T1WI signal, heterogeneous high T2WI signal; hemorrhagic foci may be visible	Low T1WI signal, high T2WI signal; “target sign” or whorled appearance may occur
PET-CT (18F-FDG) metabolic activity	Low or mild FDG uptake (remains benign in nature even with active degeneration)	Significantly increased FDG uptake (high SUVmax, correlated with mitotic index)	Diffuse and significantly high FDG uptake due to high-grade malignancy	Moderate-to-severe increase in FDG uptake (although benign, it can mimic malignant manifestations)
Key clinical clues	History of laparoscopic uterine surgery or myoma morcellation	Upper abdominal pain, gastrointestinal bleeding, anemia; no specific medical history	Rapidly enlarging abdominal mass, weight loss, advanced age	Asymptomatic or slow-growing; mostly an incidental finding

Due to the retrospective study design and actual clinical limitations, only an enhanced CT examination was performed in this case. The MRI and PET-CT related characteristics listed in this table are standardized theoretical diagnostic criteria synthesized from the cited literature.

This case underscores the importance of medical history in the diagnostic process. A history of laparoscopic morcellation is the strongest predictor of parasitic uterine leiomyoma (20, 21). Systematic reviews by Lete et al. (7) and Gómez Portilla et al. (22) both found that the vast majority of parasitic leiomyomas are diagnosed in women with a history of prior uterine surgery. Therefore, for any female patient with a history of morcellation who presents with an abdominal mass, regardless of the tumor’s location, parasitic leiomyoma should be considered a highly suspicious diagnosis.

The treatment of parasitic uterine leiomyoma is primarily surgical, especially for cases that are symptomatic or where malignancy cannot be ruled out. Laparoscopic resection is the preferred method, but strict precautions must be taken to prevent recurrence. This includes using contained power morcellation (in-bag morcellation) to avoid the scattering of tissue fragments, a practice currently recommended as the gold standard by several gynecological associations (23). Latest studies by Aoyagi et al. (24) and Fujita et al. (25) have reconfirmed the safety and efficacy of laparoscopic surgical resection, with Fujita et al. specifically reporting a case of multiple postoperative parasitic myomas, highlighting the ongoing importance of preventing intraoperative tissue dissemination. In this case, retrieving the specimen using a collection bag was crucial to minimizing the risk of recurrent leiomyomatosis.

This case report has certain limitations. First, the follow-up period was relatively short. Although the patient’s short-term postoperative follow-up CT scan showed no abnormalities and symptoms resolved, the long-term risk of recurrence after lesion resection cannot be assessed due to the lack of long-term follow-up data, which requires further observation in future work. Second, due to the patient’s financial constraints, advanced imaging examinations such as MRI or PET-CT were not performed preoperatively, which affected the certainty of our preoperative diagnosis. Relying solely on contrast-enhanced CT limited our ability to perform only a simple morphological characteristic assessment, thereby creating a significant diagnostic blind spot. From a critical diagnostic perspective, MRI possesses superior soft-tissue resolution; for an extrauterine mass accompanied by red degeneration like in this case, MRI typically exhibits a characteristic and well-defined high T1 signal due to hemorrhagic infarction and proteinaceous fluid, contrasting sharply with the fluid-like low T1WI and high T2WI signals shown by GISTs with cystic necrosis (26). Meanwhile, PET-CT can differentiate between the two based on metabolic activity, as malignant or high-risk GISTs usually display intense FDG uptake, whereas benign leiomyomas or parasitic leiomyomas typically show low or faint metabolic uptake (27). In addition, due to objective historical reasons, detailed morcellation records from the 2019 surgery could not be obtained, although the clinical course highly suggests the possibility of iatrogenic implantation. Nevertheless, this case records detailed and complete clinicopathological data and intraoperative findings, providing an important reference value for improving clinicians’ ability to differentially diagnose incidental mesenteric masses.

## 4 Conclusion

Parasitic uterine leiomyoma is a rare but increasingly recognized complication of laparoscopic uterine surgery. This case illustrates the diagnostic pitfall of misdiagnosing a mesenteric parasitic myoma as a primary gastrointestinal tumor such as a GIST. Clinicians must maintain a high level of suspicion for female patients with a history of uterine myomectomy who present with an abdominal mass. Preoperative recognition helps in formulating an appropriate surgical plan and avoiding unnecessary radical bowel resection.

## Data Availability

The original contributions presented in the study are included in the article/supplementary material. Further inquiries can be directed to the corresponding authors.
